# Brasiliensin: A novel intestinal thrombin inhibitor from *Triatoma brasiliensis* (Hemiptera: Reduviidae) with an important role in blood intake

**DOI:** 10.1016/j.ijpara.2007.04.017

**Published:** 2007-10

**Authors:** R.N. Araujo, I.T.N. Campos, A.S. Tanaka, A. Santos, N.F. Gontijo, M.J. Lehane, M.H. Pereira

**Affiliations:** a Departamento de Parasitologia, Instituto de Ciências Biológicas, UFMG, Bloco 14, Sala 177, Av. Antônio Carlos 6627, Belo Horizonte, MG, Brazil; b Departamento de Bioquímica, Escola Paulista de Medicina, UNIFESP-EPM, São Paulo, SP, Brazil; c Liverpool School of Tropical Medicine, Pembroke Place, Liverpool L3 5QA, UK

**Keywords:** Triatomine, *Triatoma brasiliensis*, RNA interference, Brasiliensin, Feeding efficiency, Thrombin inhibitor, Intestinal anti-coagulant

## Abstract

Every hematophagous invertebrate studied to date produces at least one inhibitor of coagulation. Among these, thrombin inhibitors have most frequently been isolated. In order to study the thrombin inhibitor from *Triatoma brasiliensis* and its biological significance for the bug, we sequenced the corresponding gene and evaluated its biological function. The *T. brasiliensis* intestinal thrombin inhibitor, termed brasiliensin, was sequenced and primers were designed to synthesize double strand RNA (dsRNA). Gene knockdown (RNAi) was induced by two injections of 15 μg of dsRNA into fourth instar nymphs. Forty-eight hours after the second injection, bugs from each group were allowed to feed on hamsters. PCR results showed that injections of dsRNA reduced brasiliensin expression in the anterior midgut by approximately 71% in knockdown nymphs when compared with controls. The reduction in gene expression was confirmed by the thrombin inhibitory activity assay and the citrated plasma coagulation time assay which showed activity reductions of ∼18- and ∼3.5-fold, respectively. Knockdown nymphs ingested approximately 39% less blood than controls. In order to confirm the importance of brasiliensin in blood ingestion, fourth instar nymphs were allowed to ingest feeding solution alone or feeding solution containing 15 U of thrombin prior to blood feeding. Fifty-five percent less blood was ingested by nymphs which were fed thrombin prior to blood feeding. The results suggest that anticoagulant activity in the midgut is an important determinant of the amount of blood taken from the host. The role of anticoagulants during blood ingestion is discussed in the light of this novel insight.

## 1 Introduction

Every hematophagous invertebrate studied to date produces at least one inhibitor of coagulation in the salivary glands and/or intestine. Most of these target one or more of the serine proteases which make up the tissue factor pathway, e.g. factor VIIa, factor Xa and/or thrombin (Ledizet et al., 2005). Among them, thrombin inhibitors have frequently been isolated from arthropods. For example, the protein Amblin from the ixodid tick *Amblyomma hebraeum* (Lai et al., 2004), ornithodorin from the soft tick *Ornithodorus moubata* (van de Locht et al., 1996) and the major salivary thrombin inhibitor from *Glossina morsitans* that is also expressed in the midgut (Cappello et al., 1998).

In triatomines, thrombin inhibition was demonstrated in the saliva (Noeske-Jungblut et al., 1995) and also seen for the intestinal rhodniin from *Rhodnius prolixus* (Friedrich et al., 1993), dipetalogastin from *Dipetalogaster maximus* (Mende et al., 1999) and infestin from *Triatoma infestans* (Campos et al., 2002). Infestin was found in the anterior midgut and is encoded by a unique gene incorporating seven Kazal type domains.

*Triatoma brasiliensis* are vessel feeding, hematophagous arthropods and one of the main Brazilian vectors of *Trypanosoma cruzi*, the causative agent of Chagas disease. They take very large blood meals (up to 370 μl for the fifth instar nymph) which can take more than 35 min to ingest (Guarneri et al., 2003). In order to control the hemostatic system of their hosts and guarantee successful feeding and management of the ingested blood, triatomines produce several anti-hemostatic substances in their saliva and intestine. During the feeding process, saliva is released the whole time and some of it is ingested with the blood (Soares et al., 2006). The undigested blood is then stored in the wide anterior part of the midgut, in which water and ions are transported to the haemolymph and malpighian tubules, and excreted via the rectum. The concentrated blood is passed in small amounts into the digestive and absorptive part of the posterior midgut (Kollien and Schaub, 2000). It has been verified in ixodid ticks that the ingestion of blood decreased when the salivary anticoagulant was depressed (Narasimhan et al., 2004). In contrast, when salivary glands were removed from *R. prolixus*, although they pierced host skin more often than normal bugs, if they are exposed to the host for sufficient time then the salivarectomy did not affect the amount of blood ingested (Ribeiro and Garcia, 1981). Here we have investigated one aspect of this apparent contradiction. As part of a gene discovery program for *T. brasiliensis*, we have found an orthologue of the anti-thrombin gene infestin from *T. infestans*, which was termed brasiliensin. In this paper, we describe this gene and use RNA interference (RNAi) to knock down brasiliensin (Araujo et al., 2006) to determine its physiological role during blood ingestion by *T. brasiliensis*.

## 2 Materials and methods

### 2.1 Triatomine bugs

*Triatoma brasiliensis* were reared under controlled temperature (26 ± 2.0 °C) and humidity (65 ± 5.0%), 12/12 light/dark and fed weekly on chickens or rats. The fourth instar specimens used in the experiments had similar physiological status (7 ± 1 days after molt).

### 2.2 Brasiliensin gene cloning and sequencing

Total RNA was extracted from the anterior midgut of four *T. brasiliensis* using Trizol solution (Invitrogen) according to the manufacturer’s instructions. First strand cDNA was synthesized from 1.25 μg of total RNA with Improm II (Promega) and d(T)12 following the manufacturer’s instructions. First strand cDNA was used as a template in a PCR performed with primers designed from the infestin gene of *T. infestans* (Lovato et al., 2006). PCR product was analysed by electrophoresis in 1% agarose gel and the desired amplicom was cloned into the pGEM-T Easy vector. The complete gene sequence was determined on an ABI Prism 377 DNA sequencer with DYEnamicTM ET Terminator Cycle Sequencing Kit (GE Healthcare Life Sciences).

### 2.3 Double strand RNA synthesis

Brasiliensin cDNA was amplified by PCR using specific primers (forward 5′-gagttctacaccgggtttgc-3′ and reverse 5′-ccatctgaaccacacactgg-3′, annealing temperature (*T*_a_) = 60 °C) conjugated with 23 bases of the T7 RNA polymerase promoter. PCR was carried out for 35 cycles (94 °C for 30 s, 60 °C for 30 s, 45 °C for 45 s) with 1 μl of the cDNA in addition to 200 nM of each primer, 200 μM deoxyribonucleotide triphosphate (dNTP) and 1 U *Taq* Phoneutria (Phoneutria, Brazil) in a final volume of 20 μl. The 575 bp PCR products, 529 bp of the brasiliensin and 46 bp of the T7 promoter sequences, were used as a template for double-stranded RNA (dsRNA) synthesis using the T7 Ribomax Express RNAi System (Promega). After synthesis, the dsRNA was isopropanol-precipitated, resuspended in ultra pure water and quantified by 260 nm wavelength spectrophotometry. The quality of the dsRNA products was verified by agarose gel electrophoresis. The dsRNA was kept at −80 °C until use.

### 2.4 Delivery of dsRNA

Fourth instar nymphs were injected once or twice laterally into the thoracic haemocoel with a 48-h interval between injections. Each bug from the knockdown group was injected with 15 μg brasiliensin dsRNA diluted in 2 μl of 0.9% NaCl saline solution (brasiliensin dsRNA group) while each bug from the control groups received 2 μl of saline alone (saline control group) or 2 μl of saline containing 15 μg dsRNA from the β-lactamase gene (BLA dsRNA group). Forty-eight hours after the second injection, nymphs were fed on hamsters (Araujo et al., 2006).

### 2.5 Verification of knockdown by PCR

RNA was extracted from anterior midguts of individual nymphs from each group 48 h after dsRNA injection and semi-quantitatively assessed by cDNA synthesis and PCR for the level of gene knockdown. PCR was performed using primers for brasiliensin (as in section 2.3.) and the 18s rRNA (RP18s: forward 5′-cctgcggcttaatttgactc-3′ and reverse 5′-gtacaaagggcagggacgta-3′, *T*_a_ = 60 °C) as a loading control. PCR was carried out as above, but for 23 cycles. The products were analysed by 2% agarose gel electrophoresis and the intensity of bands was measured by densitometry using the Alpha DigiDoc 1201™ software (Alpha Innotech).

### 2.6 Detection of thrombin inhibitory activity

Anterior midgut homogenates were prepared for each insect 48 h after the last dsRNA injection. Insects were dissected and their anterior midguts were removed and homogenized in 100 μl of 100 mM Tris–HCl, pH 8.0, containing 150 mM NaCl and 0.01% Triton X-100. Homogenates were centrifuged at 15,800*g* for 10 min and supernatants were used in inhibitory assays towards human thrombin (Enzyme Systems). For each assay, different volumes of homogenate were incubated with 0.03 U (generates a Δ*A*_405_ = 0.050/min) human thrombin for 5 min at 37 °C. After incubation, the chromogenic substrate H-D-Phe-Pip-Arg-*p*-nitroanilide (Chromogenix) was added to a final concentration of 125 μM. Substrate hydrolysis was monitored at 405 nm. The total protein of each homogenate was measured with DC Protein Assay (Bio-Rad) using BSA as a control (Bradford, 1976). Results were expressed as a percentage of homogenate necessary to inhibit 50% of the enzyme activity.

### 2.7 Citrated plasma recalcification time assay

Anterior midguts were dissected and transferred to ice-cold microcentrifuge tubes containing 30 μl *N*-2-hydroxyethylpiperazine-*N*′-2-ethanesulfonic acid (Hepes)/NaCl buffer (20 mM Hepes–100 mM NaCl, pH 7.5). Samples were homogenized and centrifuged for 3 min at 12,000*g*. The supernatant was transferred to a new tube and diluted in Hepes/NaCl buffer to 1:8. For the assay, 30 μl of the diluted samples were added in 96-well plates and incubated with 30 μl citrated human plasma (0.38% trisodium citrate) at 37 °C for 5 min. After incubation, coagulation was triggered by the addition of 30 μl of 25 mM CaCl_2_ pre-warmed to 37 °C. The reaction was performed at 37 °C and the increase in turbidity was monitored at 655 nm every 10 s. The coagulation time value was defined as the time taken for the turbidity to achieve an absorbance reading of 0.025 absorbance units, the absorbance at zero time being taken as zero absorbance units (Ribeiro, 2000).

### 2.8 Blood intake after thrombin ingestion

Fourth instar nymphs were fed with feeding solution alone (125 mM NaCl, 30 mM KCl, 2 mM MgCl_2_, 1 mM CaCl_2_, 5 mM NaHCO_3_, 2 mM NaH_2_PO_4_, 1 mM glucose, and 1 mM ATP, pH 7.4) or feeding solution containing 15 U human thrombin (Sigma). Nymphs were allowed to ingest approximately 20 μl of the experimental feeding solution and immediately after ingestion they were allowed to blood feed to repletion on hamsters. The size of the blood intake was calculated by subtracting bug weight after ingestion of the feeding solution from the final weight (after ingestion of blood).

### 2.9 Data and statistical analysis

The results from all experiments are shown as the mean ± standard error (SEM). All Statistical analysis was performed with GraphPad Instat TM for windows. Analysis of variance (ANOVA) was used to verify differences between groups. Student’s *t* test was used to test for differences measured between two variables. *P* < 0.05 was considered significant.

## 3 Results

### 3.1 Sequence of brasiliensin

**Fig. 1:**
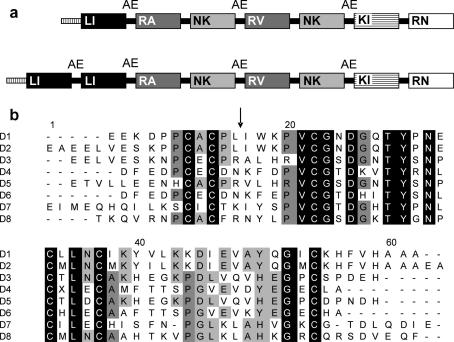
Diagram of the full-length brasiliensin sequence and amino-acid comparison of the eight domains. (a) Representation of the infestin gene (top) in comparison to the *Triatoma brasiliensis* brasiliensin gene (bottom). Each box represents a domain. Domains with high similarity are the same color. Letters inside the boxes represent the amino acids at the P1 position of the reactive sites (arrow in b). Inter domain loops containing AE residues are indicated. The box with vertical lines at the beginning of each gene represents the signal peptide. (b) Alignment of the translated domains (D1–D8) present in the *T. brasiliensis* Kazal type serine protease inhibitor gene. Alignment was performed with Clustal W (Thompson et al., 1994). Identical amino acid residues in all domains are black boxed. Dark gray and light gray indicate amino acids present in at least five and four domains, respectively. The arrow indicates the putative reactive site determined by comparison with other Kazal type inhibitors.

The brasiliensin gene (GenBank Accession No. DQ915949) is a multi domain serine protease inhibitor similar to rhodniin, dipetalogastin and infestin. The gene codes for a protein composed of eight Kazal type domains (Fig. 1) that it is believed to be processed further to generate small inhibitors composed of one or two domains. The presence of the residues Ala-Glu in the interdomain loop may indicate the cleavage points of the mature inhibitors, as inferred by similarities with infestin. If that is the case, the first two N-terminal peptides (brasiliensin domain 1 and brasiliensin domain 2) each contain a putative single elastase inhibitor domain. The next three putative peptides each contain two different pairs of inhibitor domains and thus form dual head inhibitors. Brasiliensin domains 3–4 and brasiliensin domains 5–6 each contain two thrombin inhibitor domains. The final putative peptide, brasiliensin domains 7–8, contains a factor XIIa inhibitor domain (Campos et al., 2004; Lovato et al., 2006).

### 3.2 Brasiliensin knockdown

**Fig. 2:**
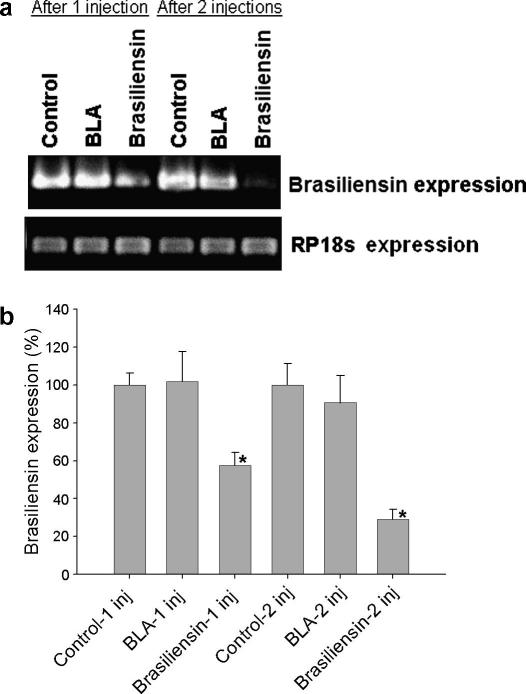
PCR measurement of brasiliensin mRNA knockdown from a fourth-instar nymph anterior midgut. The housekeeping 18s rRNA (RP18s) was used as a loading control. (a) Shows the reduction in brasiliensin expression from one insect after one (columns 1–3) or two (columns 4–6) injections. (b) Average and standard error from the PCRs using individual anterior midguts from insects (*n* = 4). Asterisks indicate the groups with significant statistical difference (*P* < 0.05). Control, saline injected groups; BLA, β-lactamase gene double-stranded RNA (dsRNA) injected group; Brasiliensin, brasiliensin dsRNA injected group.

The effect of RNAi on gene knockdown was verified by PCR. Inhibition could be observed 48 h after the first injection of dsRNA. PCR performed 48 h after one injection of 15 μg brasiliensin dsRNA significantly reduced gene expression by 42 ± 14% (*P* = 0.0149) while for two injections the reduction was 71 ± 11% (*P* = 0.005) 48 h after the second injection, when compared with control groups. The group injected with non-specific dsRNA (BLA group) had no significant reduction in brasiliensin expression when compared with saline-injected controls (*P* > 0.05) (Fig. 2).

**Fig. 3:**
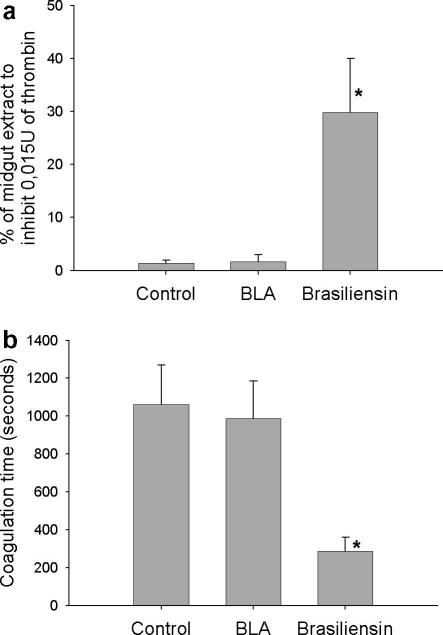
Activity assays performed with extracts from the anterior midgut of *Triatoma brasiliensis* from the saline injected group (control), β-lactamase gene double-stranded RNA (dsRNA) injected group (BLA) and brasiliensin dsRNA injected group (Brasiliensin) extracted 48 h after the second injection (*n* = 6). (a) Thrombin activity assay. Extracts were assayed using 0.03 U human thrombin and the synthetic chromogenic substrate S2238 (0.125 mM). Results were expressed as the percentage of homogenate necessary to inhibit 50% of the enzyme. (b) Citrated plasma recalcification time assay. Coagulation time was defined as the time taken for the turbidity to achieve an absorbance reading of 0.025 absorbance units, the absorbance at zero time being taken as zero absorbance units. Asterisks indicate groups with significant statistical differences (*P* < 0.05). Results are represented as the average ± standard error.

The reduction of brasiliensin expression was confirmed by functional assays of thrombin inhibitory activity and citrated plasma recalcification time assays using anterior midgut extracts. In control bugs, the average amount of intestine extract necessary to inhibit the same amount of thrombin was considerably lower (approximately 18-fold) compared with knockdown bugs (Fig. 3a). Bugs from the saline-injected and BLA dsRNA-injected groups needed 1.4 ± 0.51% and 1.6 ± 1.3% of the intestine extract to inhibit 0.015 U thrombin, respectively, while the knockdown group needed 29.8 ± 10.2% of the intestine extract. The difference between knockdown and control groups was statistically significant (*P* = 0.0132).

Brasiliensin RNAi also affected intestinal anti-clotting activity (Fig. 3b). Anterior midgut extracts from bugs tested in the citrated plasma recalcification time assay showed that the anticoagulant activity of brasiliensin dsRNA group midgut extracts was significantly lower than control bugs (*P* = 0.009). The brasiliensin dsRNA extracts prolonged the initiation of coagulation approximately 3.5 times less than the saline control and BLA dsRNA groups.

### 3.3 Anticoagulant activity in the anterior midgut

The citrated plasma recalcification time assay was performed using extracts from separate component parts of *T. brasiliensis* anterior midguts dissected from starved bugs 20 days after the last molt. Three separate homogenates of the anterior midgut were analysed – the contents, the midgut wall and the whole anterior midgut (contents plus wall). The coagulation time for the whole intestine was 1.051 ± 187 s, for the contents it was 891 ± 169 s and for gut wall alone it was 152 ± 28 s (Table 1).

### 3.4 Effect of brasiliensin knockdown on bug feeding

**Fig. 4:**
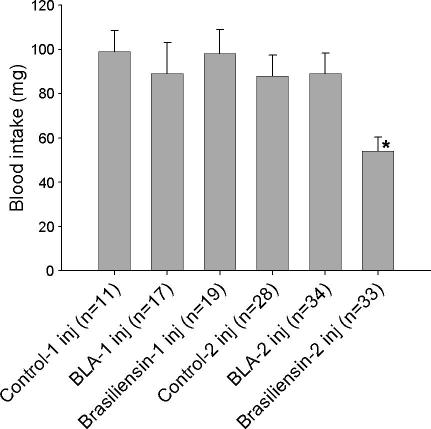
Blood intake of fourth instar nymphs from the saline injected group (control), β-lactamase gene double-stranded RNA (dsRNA) injected group (BLA) and brasiliensin dsRNA injected group (Brasiliensin) after one (columns 1–3) or two (columns 4–6) injections. The nymphs were allowed to feed on hamsters 48 h after the final injection. Asterisks indicate groups with significant statistical differences (*P* < 0.05). Results are represented as the average ± standard error.

To assess the importance of brasiliensin in the feeding process, blood intake of bugs from the brasiliensin knockdown group was compared with the control groups. Nymphs were allowed to feed on hamsters *ad libitum* 48 h after the last injection. Results showed that there is no difference in blood ingestion between knockdown and controls after one injection. However, when two injections were given, brasiliensin knockdown bugs ingested significantly lower amounts of blood compared with controls (*P* = 0.005). Their average blood intake was 54.2 ± 6.4 mg while that of the saline control group was 88.9 ± 9.3 and that of the BLA dsRNA-injected group was 87.7 ± 9.5 mg (Fig. 4).

**Fig. 5:**
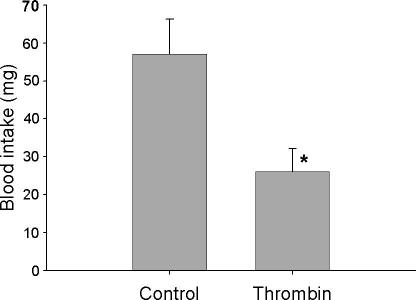
Blood intake of fourth instar nymphs after ingestion of 20 μl feeding solution alone (control, *n* = 22) or containing 15 U human thrombin (thrombin, *n* = 25). The nymphs were allowed to feed on hamsters immediately after ingesting the feeding solution. Asterisks indicate significant statistical differences (*P* < 0.05) between groups. Results are represented as the average ± standard error.

The importance of intestinal anticoagulant activity during blood feeding was confirmed by observing blood ingestion in bugs that had previously ingested thrombin. Fourth instar nymphs previously fed with 20 μl of feeding solution (control) took blood meals of 57 ± 9 mg (Fig. 5). Ingestion of blood was significantly reduced in the group previously fed with 20 μl of feeding solution containing 15 U thrombin (thrombin group) which took blood meals of only 26 ± 6 mg. This 55% reduction in blood intake of nymphs that ingested thrombin was statistically significant (*P* = 0.0066). The total weight gain (20 mg feeding solution + blood intake) from nymphs was ∼77 mg for the control group and ∼46 mg for the thrombin group, which is equivalent to the weight gain of nymphs submitted to RNAi experiments that only ingested blood (Fig. 4).

After dissection, clots were observed in the anterior midgut of bugs from the knockdown group and from those that ingested feeding solution with thrombin prior to blood feeding. In the control group, the blood was apparently only compacted and in some cases with small clots, which was disrupted easily by shaking (data not shown).

## 4 Discussion

It has been described for more than a century that hematophagous invertebrates produce potent inhibitors of thrombosis, presumably to facilitate the taking of a blood meal (Ledizet et al., 2005). Here, we describe the sequence of the *T. brasiliensis* intestinal thrombin inhibitor brasiliensin, its role in inhibition of blood clotting in the midgut and the importance of this phenomenon for the intake of blood by the bugs. Our data show a novel role of the insect’s antithrombotic armoury.

The brasiliensin gene in *T. brasiliensis* has high identity to the sequence of the infestin gene from *T. infestans*, with the exception of the additional first domain at the 5′ end of the gene. This domain is almost identical to the second domain and is probably a result of exon duplication. Exon duplication is commonly used as an explanation for the generation of multiple domains in many Kazal type inhibitors (Scott et al., 1987). Since its reactive site is identical to the reactive site of the second domain, it is probably also a subtilisin A, neutrophil elastase and chymotrypsin inhibitor (Campos et al., 2004; Lovato et al., 2006). As observed for the infestin gene in *T. infestans*, the sequence Ala-Glu is present in the loops that are between domains and are cleaved after some still unknown process that generates the inhibitors found in the gut.

RNAi has become a valuable tool for investigating gene function in eukaryotes. The mechanism, triggered by dsRNA (>30 bp) or small interfering RNAs (siRNA) with only 19-23 bp, cleaves the cognate mRNA and inhibits the translation of the gene (Mello and Conte, 2004). The possibility of using RNAi in triatomine bugs was previously demonstrated for *R. prolixus* salivary gland genes (Araujo et al., 2006). In this study, we showed that it is also effective for the knockdown of intestinal genes of *T. brasiliensis*. Although the dsRNA used to knock down the brasiliensin has 529 bp ranging from the fifth to the eightieth domain of the molecule, the RNAi mechanism implies that the whole mRNA was degraded, with no translation of any of the eighth domains. The reduction in expression was observed as early as 48 h after one injection of dsRNA and the level of knockdown was enhanced after two injections, reaching approximately 71% of the intestinal brasiliensin mRNA. The reduction in mRNA expression correlates with the reduction in the thrombin inhibitory activity assay using a specific chromogenic substrate (∼18-fold reduction). In addition to the high similarity with infestin, this result reinforces the suggestion that brasiliensin has thrombin inhibitory activity.

Reduction in the anticoagulant activity from knockdown midgut extracts was also observed using the citrated plasma recalcification assay (∼3.5-fold reduction). This assay (that detects alterations in several points of the coagulation cascade) was used because the brasiliensin amino acid sequence is very similar to infestin from *T. infestans*, which also inhibits factors XIIa and Xa (Campos et al., 2004). The reduction of anticoagulant activity observed in these assays could be verified in knockdown insects *in vivo*, in which clots were found inside the anterior midguts.

No difference was observed in the amount of blood ingested between controls and insects from the knockdown group after only one injection of dsRNA or insects that ingested small amounts of exogenous thrombin (data not shown). Reduction of blood ingestion, as seen in bugs after two injections of brasiliensin dsRNA, was only verified in nymphs after ingestion of high amounts of thrombin (∼15 U). In *T. infestans*, native purified infestin strongly inhibited thrombin with a dissociation constant (*K*_i_) of 43.5 ± 31.5 pM (Campos et al., 2002). Brasiliensin might have a similar mechanism. It is probable that clot formation in insects that ingested exogenous thrombin occurred because of the large quantity of thrombin ingested, which depleted most of the brasiliensin.

Anticoagulant activity could be found in the *T. brasiliensis* anterior midgut even in bugs starved for 20 days. Most of the activity was found in the contents of the midgut and just a small amount of activity was associated with the anterior midgut wall, suggesting that the anticoagulant molecules are synthesized and stocked in the anterior midgut lumen prior to the ingestion of blood. The reduction of the intestinal anticoagulant activity observed a few days (4 days after the first or 2 days after the second injection) after the dsRNA injections indicates that the half-life of the brasiliensin in the midgut is short, suggesting that the insects need a constant turnover of brasiliensin. Although the constant production of brasiliensin seems uneconomical for the insects, it is necessary because the intestine environment must be constantly ready to receive ingested blood, as triatomine bugs do not know when they will find a host.

Salivary anticoagulant activity has been detected in all triatomines studied to date, showing quantitative (Ribeiro et al., 1998) and qualitative (Pereira et al., 1996) variation between species. Considering that part of the saliva is ingested during the feeding process in triatomines, the anterior midgut activity is probably the cumulative result of the salivary and intestinal anticoagulants. The clots found in brasiliensin knockdown insects in this work showed that salivary (and other intestinal, if there are any) anticoagulants were not able to prevent the blood clotting in the midgut; this demonstrates the important role of brasiliensin in avoiding coagulation *in vivo*.

Feeding efficiency of triatomine bugs on rats correlated significantly with the amount of apyrase activity as well as with vasodilatory activity, but not with the salivary anti-clotting activity (Ribeiro et al., 1998). The feeding process of the bugs may explain the minor role of the anticoagulant activity for obtaining blood from vertebrate skin. Triatomines are vessel feeders that introduce their terminal mouthparts directly into the vessel lumen (arterioles or venules) (Lavoipierre et al., 1959). This process minimises the contact with tissue factors that trigger the extrinsic clotting pathway, probably diminishing the importance of anti-clotting factors present in saliva. In addition, coagulation has a minor role in small vessel hemostasis (Ribeiro, 1987). Salivary anti-clotting factors should be much more important for pool feeders because in this case the blood is exposed intimately to the tissue factors in the wound produced by the insect mouthparts in the host skin.

Our results show that in insects where blood coagulation in the anterior midgut was permitted by knockdown of midgut-specific anticoagulants or by administration of exogenous thrombin, blood intake was considerably reduced. The presence of clots in the midgut does not appear to be a major problem for the digestion process as insects from both the knockdown and the thrombin-ingested group were able to digest blood, feed again and molt to the next instar (data not shown). Clots may have been broken down by an intestinal fibrinolitic activity similar to that previously described for *R. prolixus* (Hellmann and Hawkins, 1964).

The simplest explanation for the reduction in the volume of blood ingested by knockdown insects and those ingesting exogenous thrombin is that blood in the midgut must remain in a liquid state during feeding and backpressure, induced by increased viscosity, will prevent successful pumping of blood into the midgut. Once the blood coagulation cascade is initiated, there is a rapid increase in viscosity that occurs during the clotting process (Puckett et al., 2005). Therefore, avoiding the increase in blood viscosity in the digestive tube in hematophagous insects during the blood-pumping phase seems to be an important physiological function for intestinal anticoagulants. Besides coagulation, other mechanisms such as platelet aggregation can influence blood viscosity. As thrombin is also a potent activator of platelet aggregation (Ribeiro and Francischetti, 2003), it is possible that the reduction in brasiliensin in knockdown insects elicited platelet aggregation that could have helped in clot formation in the intestine.

Anticoagulants may also have a role in parasite-vector interactions. After entering the anterior midgut, *T. cruzi* are confronted with a range of potentially harmful molecules, mostly arising from the saliva (Kollien and Schaub, 2000). It remains to be seen whether anticoagulant activity would improve the environment for trypanosomes by preventing the immobilisation of the ingested parasites and permitting them to move throughout the midgut (Cappello et al., 1998; Friedrich et al., 1993).

The role of anticoagulants during blood ingestion proposed here is a novel insight. Differences in the capability of inhibition of the coagulation process inside the digestive tube could be also important in explaining the varied feeding performance observed in triatomine bugs (Pereira et al., 2006). A previous study using an artificial feeding apparatus showed that an increase in the viscosity of the diet caused a decline in both the frequency of the cibarial pump and the average stroke volume of the pump (Smith, 1979). A better understanding of this physiological function in hematophagous insects can contribute to knowledge of its feeding and digestive processes, interaction with vertebrate hosts and parameters involved in interaction with trypanosomatids.

## Figures and Tables

**Table 1:** Citrated plasma recalcification time assay from an extract of anterior midgut contents (AMC), anterior midgut wall (AMW) or the whole anterior midgut (AMC + AMW) of fourth instar nymphs starved for 20 days

	AMC	AMW	AMC + AMW
Coagulation time (seconds, average ± SEM.)	891 ± 169	152 ± 28	1.051 ± 187
Number of nymphs	9	9	9

Coagulation time was defined as the time taken for the turbidity to achieve an absorbance reading of 0.025 absorbance units, the absorbance at zero time being taken as zero absorbance units.
